# Supplementary material for: Navigating HIV self-testing: Concerns among adolescents and young people aged 15–24 years in Uganda. An exploratory qualitative study
Source: PLoS One. 2026 Feb 5;21(2):e0330000. doi: 10.1371/journal.pone.0330000 (PMC12875464; doi:10.1371/journal.pone.0330000)
Supplement: S1 File — (ZIP) [file pone.0330000.s001.zip › Interview Guides/FGD guide.docx]

**Interview - Group Discussion guide for HIVST for AYP**

1. What are the current HIV testing services available in this community?

***Probe*** *what is the perception and level of acceptance and use of the existing services by adolescents and young people (AYP)?*

1. Please share with me what you know or have heard about HIVST and HIVST procedures. ***Probe*** *where they heard it from, their thoughts about HIVST*
2. What are your views about HIVST? ***Probe*** *for comparison between current routine HIV testing and HIVST?*
3. When made available, where and how do you think HIVST kits should be accessed and distributed? ***Probe*** *for location i.e. facilities, vendors, peers, clinic, pharmacy, mobile clinics and community centres.*

***Probe*** *for reasons for the selected point of dissemination. Probe for person to disseminate.*

*Where would you recommend a friend to get HIVST kits?* ***Probe*** *Why?*

1. *What are your feelings about receiving them from peers or using a peer to peer distribution model?* ***Probe*** *best ways to share information among peers and adolescent and the facilitators and barriers of using peer distributors*
2. Would you be willing to pay for the Kit? How much would you be willing to pay for it and why? ***Probe*** *what would motivate you to pay?*
3. What would be the possible challenges associated with using and distributing HIV self-test kits?
4. What aspects would encourage you to take up HIVST? ***Probe*** Potential for a dramatic increase in knowledge of HIV status, increased confidentiality, increased convenience, Autonomy and empowerment).
5. What are your concerns about HIVST?

***Probe*** *Greater potential for inaccurate results, Psychological danger when decoupling testing and counselling, Greater difficulty ensuring referral to treatment and care, Potential unethical use of HIV self-testing, Self-testing as justification for unprotected sex, Concern for safe disposal of biohazard materials)*

1. [Demonstrate for participant the use of the OraQuick kit] then ask - What is your comment on the design of the kit and the testing procedures? ***Probe*** *if this is something that would be acceptable to AYP.*
2. What are your perception /opinions on test accuracy? ***Probe*** *How would you perceive the results, what would you do if the result was negative positive, unclear or you are uncertain?*
3. Is there anything else you would love to share with me about what we have been discussing or HIV testing in general?

**Thank you for accepting to participate in this research study**
