# Supplementary material for: Navigating HIV self-testing: Concerns among adolescents and young people aged 15–24 years in Uganda. An exploratory qualitative study
Source: PLoS One. 2026 Feb 5;21(2):e0330000. doi: 10.1371/journal.pone.0330000 (PMC12875464; doi:10.1371/journal.pone.0330000)
Supplement: S1 File — (ZIP) [file pone.0330000.s001.zip › Interview Guides/IDI Interview Guide.docx]

**HIV Self-testing among young people: Assessing the knowledge, acceptability and social implications of a peer-to-peer HIVST distribution model among adolescents aged 15 to 24 in Zambia and Uganda.**

**(HISTAZU)**

| **Qualitative Interview Guide** |
| --- |
| **Background information**   - Please tell me a little bit about yourself? (hobbies, friends, where you hangout for fun) - Tell me about the last time you tested for HIV. Why did you decide to test for HIV? Where did you test for HIV? What was your experience of testing for HIV? If NEVER tested for HIV. What are the reasons you have never tested for HIV? |
| **HIV RISK PERCEPTIONS**   - How do you think young people that you know are at risk of contracting HIV? Probe: which group of people do you think are more at risk: sex, gender, age?​ - How do you think you are/ are not at risk of contracting HIV? - Please tell me about ways that young people (including yourself) are using to protect against HIV. |
| **Perceptions and motivations of self-testing**   - What are your opinion about self-testing? Probe for where to get the kits, price of the kits, ability to read the correct results, who to distribute them, feasibility of young people adapting self-testing - Please share with some of the perceptions that other young people like you have about self-testing - What are some of the motivations/facilitators of self-testing? - Please share with me some of the barriers to self-testing among young people. Probe for counselling needs, fear of the needle prick…. - How can these barriers be overcome? |
